# Involvement of Indoleamine 2,3-Dioxygenase in Impairing Tumor-Infiltrating CD8^+^ T-Cell Functions in Esophageal Squamous Cell Carcinoma

**DOI:** 10.1155/2011/384726

**Published:** 2011-10-13

**Authors:** Ge Zhang, Wan-Li Liu, Lin Zhang, Jun-Ye Wang, Miao-Huan Kuang, Peng Liu, Yue-Hao Lin, Shu-Qin Dai, Jun Du

**Affiliations:** ^1^Department of Microbial and Biochemical Pharmacy, School of Pharmaceutical Sciences, Sun Yat-Sen University, Guangzhou 510006, China; ^2^State Key Laboratory of Oncology in Southern China, Sun Yat-Sen University Cancer Center, Guangzhou 510060, China; ^3^Department of Clinical Laboratory, Sun Yat-Sen University Cancer Center, Guangzhou 510060, China; ^4^Department of Thoracic Surgery, Sun Yat-Sen University Cancer Center, Guangzhou 510060, China

## Abstract

The indoleamine 2,3-dioxygenase-(IDO-) mediated microenvironment plays an important role in tumor immune escape. However, the inhibitory effects of IDO on the CD8^+^ tumour-infiltrating lymphocytes (CD8^+^ TILs) in esophageal squamous cell carcinoma (ESCC) have not been clarified yet. Here, we found that the level of IDO expression in ESCC tumor specimens correlated with a reduction in the number of CD8^+^ TILs. Patients with high IDO expression and a low number of CD8^+^ TILs had significantly impaired overall survival time. IDO expression and functional enzyme activity in ESCC cell lines could be induced by IFN**γ**. When exposed to the milieu generated by IDO-expressing Eca109 cells, the CD8^+^ TILs were suppressed in proliferation, and their cytolytic functions against target tumor cells were lost. These results suggested that impairing CD8^+^ TIL functions by IDO expressed in ESCC possibly contributed to the finding that patients with higher IDO expression have more aggressive disease progression and shorter overall survival time.

## 1. Introduction

Effector CD8^+^ tumor-infiltrating lymphocytes (CD8^+^ TILs) are major mediators for host's antitumor immunity [1–5]. Increasing studies have reported that higher numbers of CD8^+^ TILs within either esophageal squamous cell carcinoma (ESCC) epithelium or stroma have a better prognosis [6, 7]. Recently, several reports showed that tumoral indoleamine 2,3-dioxygenase (IDO) expression correlated with a reduced number of CD8^+^ or CD3^+^ TILs in colorectal cancer, ovarian cancer, and endometrial cancer, possibly contributing to disease progression and impaired clinical outcome [8–10]. 

IDO is responsible for initiating the first rate-limiting step in tryptophan (Trp) metabolism in the kynurenine (Kyn) pathway [11, 12]. Growing evidence suggests that various types of human tumor cells express IDO, and inflammatory mediators, especially interferon-*γ* (IFN*γ*), have the specific ability to induce IDO expression [13, 14]. Tumoral IDO expression has been shown to correlate with poor clinical prognosis in ovarian carcinoma, endometrial carcinoma, lung cancer, osteosarcoma, and colon carcinoma [8, 15–18]. IDO-mediated Trp metabolism in antigen-presenting cells and tumor cells represents a vital mechanism for potential T-cell suppression during tumor growth [19–21]. In the experimental rat lung allograft model, IDO not only reduced the number of CD8^+^ T cell infiltration but also impaired the cytotoxic function of effector CD8^+^ T-cells, this impairment was responsible for the IDO-dependent immune suppression [22]. Our previous study also showed that exposure to the milieu created by an IDO-positive nasopharyngeal carcinoma cell line significantly impaired the lymphocyte cytotoxicity against target tumor cells [23]. 

A previous study on a small group of ESCC patients showed that IDO mRNA was expressed in ESCC tumor specimens, and ESCC patients with higher levels of IDO mRNA expression had a worse survival rate than those with lower levels of IDO mRNA expression [24]. In contrast, Liu reported that the level of IDO expression did not correlate with the clinic outcomes of ESCC patients [25]. In the current study, we investigated the relationship between IDO expression and the degree of tumor infiltration of CD8^+^ T cells, and the clinical significance of IDO expression in ESCC. We also explored the effect of IDO on the proliferation and function of CD8^+^ TILs in ESCC.

## 2. Materials and Methods

### 2.1. Patients

A total of 135 ESCC samples were histologically and clinically diagnosed when the patients with primary ESCC underwent radical esophagectomy between 2001 and 2004 at the Cancer Center of Sun Yat-sen University. No patients had received prior anticancer treatment. Prior to the use of these clinical materials for investigation, informed consent from patients and approval from the Institute Research Ethics Committee were obtained. The clinical typing of the tumors was determined according to the pathological TNM classification [26]. Clinical information of the samples is described in detail in Table 1. The numbers undergoing metastasis pertain to the presence of metastasis at any time during follow-up. The median followup time for overall survival was 49.0 months for patients still alive at the time of analysis, and the time ranged from 7 to 78 months. A total of 91 (67.4%) patients died during followup.

### 2.2. Immunohistochemistry and Immunoblotting

Immunohistochemistry and western blot were performed as described previously [23, 27]. For immunohistochemistry, an anti-IDO polyclonal antibody (1 : 500, generated in our laboratory [23]) and a mouse monoclonal anti-CD8 (1 : 150, BD Pharmingen) were incubated with the tissue sections overnight at 4°C. For negative controls, the primary antibody was replaced by normal rabbit or mouse serum. After washing, tissue sections were treated with biotinylated anti-mouse or anti-rabbit antibody (Zymed), followed by further incubation with streptavidin HRP complex. For Western blot analysis, IDO was detected using an anti-IDO polyclonal antibody (1 : 5,000). An anti-*β*-actin monoclonal antibody (1 : 5,000) was used to confirm equal loading.

### 2.3. Scoring of IDO Expression in Tumor Cells

The degree of immunostaining was reviewed and scored by two independent observers, as described previously [28]. According to the percent of positive cells, one score was given for each as follows: <5% of the cells = 1 point; 6–35% of the cells = 2 points; 36–70% of the cells = 3 points; >70% of the cells = 4 points. Another score was given according to the intensity of staining as follows: negative staining = 1 point; weak staining (light yellow) = 2 points; moderate staining (yellowish brown) = 3 points; strong staining (brown) = 4 points. A final score was then calculated by multiplying the above two scores. If the final score was ≥4, the tumor was considered high expression; otherwise, the tumor was considered low expression. IDO expression in tumor stromal cells was not considered because IDO immunostaining on nontumor cells was not remarkable in all cases examined.

### 2.4. Quantification of TIL Cells within ESCC

CD8^+^ TILs were classified into two groups by their localization: (a) intraepithelial, cells infiltrating into the tumor epithelium; (b) stromal, cells infiltrating the tumor stroma adjacent to cancer epithelia or the stroma along the invasive margin of the cancer epithelia. Quantification of CD8^+^ TILs was done according to the previous reports of Cho and Schumacher with some modifications [6, 7]. Three independent areas with the most abundant CD8^+^ TIL infiltration were selected, and the intraepithelial CD8^+^ TILs and stromal CD8^+^ TILs were independently counted in each microscopic field at 200 × (0.0625 mm^2^). The average count for three areas was accepted as the number of CD8^+^ TILs in each case. We classified patients into two groups by intraepithelial CD8^+^ TIL counts: the high intraepithelial CD8^+^ TIL group (mean ≥ 10) and low intraepithelial CD8^+^TIL group (mean < 10). On the basis of stromal CD8^+^TIL counts, patients were classified into two groups in the same manner: the high stromal CD8^+^ group (mean ≥ 20) and low stromal CD8^+^ group (mean < 20).

### 2.5. ESCC Cell Culture and CD8^+^ T-Cell Isolation

The ESCC cell lines Eca109, TE-1, and KYSE140 (Cell Bank of Type Culture Collection of Chinese Academy of Sciences) were grown in RPMI 1640 (Invitrogen) supplemented with 10% fetal bovine serum. IFN*γ* (China National Biotec Group) was added to the medium at 0–500 U/mL, for the indicated time. Peripheral blood mononuclear cells (PBMCs) were isolated from whole blood of patients with ESCC before surgical treatment by Ficoll density gradient (Sigma-Aldrich). For the isolation of TILs, fresh tumor tissues from ESCC patients who underwent surgical treatment at our hospital were finely minced and subjected to enzymatic digestion. The resultant suspensions were filtered through a 25 *μ*m mesh filter, and the single-cell filtrate was washed twice in PBS followed by Ficoll/Hypaque purification. The isolation of CD8^+^ T lymphocytes from PBMCs and TILs was performed by means of immunomagnetic beads using a Dynal CD8 positive isolation kit (Invitrogen Dynal). Purity of CD8^+^ was >98% CD8^+^ as checked by flow cytometry and CD8^ +^ T cells were grown in complete RPMI 1640 medium.

### 2.6. Measurement of IDO Activity

Trp and Kyn concentrations were analyzed by reverse-phase high-performance liquid chromatography (HPLC; Waters) as described previously [23]. IDO activity was determined by the Kyn to Trp ratio (Kyn/Trp, *μ*M/*μ*M).

### 2.7. Cell Proliferation, Apoptosis, and Cytotoxicity Assay

Eca109 cells were cultured in 6-well plates (3 × 10^5^ cells/well) in the absence or presence of 50-U/mL IFN*γ* for 12 hr, and the medium was then replaced by fresh medium with or without 100 *μ*M 1-methyl-L-tryptophan (1 MT, Sigma-Aldrich). Then, 24 hr after medium replacement, the culture media were harvested as Eca109-conditioned media (Eca109-CMs). CD8^+^ T-cell proliferation was assessed by standard thymidine incorporation assay as described previously [29]. Briefly, 1 × 10^5^ CD8^+^ T cells were cultured in Eca109-CMs and stimulated with plate-bound anti-CD3 mAb (OKT3, ATCC) and soluble anti-CD28 mAb (BD Bioscience). After 72 hours of culture, 1-*μ*Ci ^3^[H] thymidine was added, and incorporation was measured after 24 hr in a *β*-Counter (Wallac). The proportion of apoptotic cells from different culture conditions were examined by flow cytometry using an Annexin V-FITC apoptosis detection kit (Beckman Coulter). 

The cytotoxic activity of CD8^+^  T cells was determined by a standard lactate dehydrogenase (LDH) release assay using the CytoTox 96 (Promega) as previously described [23]. Briefly, the Eca109 cells (5 × 10^3^ cells/well) were cultured as target cells. IL-2-stimulated CD8^+^ T cells were incubated in different Eca109-CMs as the treated effector cells. The target cells and effector cells suspensions were cocultured at various indicated effector : target (E/T) ratios. After 4 h of incubation, the release of LDH into the supernatant was quantified by recording the absorbance at 490 nm. The percentage of cytotoxicity was calculated as manufacturer described.

### 2.8. Statistical Analysis

All statistical analyses were conducted using the SPSS 16.0 statistical software package. Parametrically distributed data are presented as mean ± SD. Comparison of the number of CD8^+^ TILs in the IDO-low group and IDO-high group was performed with the Mann-Whitney *U* test. The Pearson *χ* ^2^ and Fisher's exact test were used to analyze the relationship between IDO expression and clinicopathologic characteristics or the number of CD8^+^ TILs. Survival curves were plotted by the Kaplan-Meier method and compared by the log-rank test. Comparison between paired or unpaired groups was performed using the appropriate Student's* t-*test. A *P *value of < 0.05 in all cases was considered statistically significant.

## 3. Results

### 3.1. Expression of IDO in Archival Esophageal Tumor Tissues and Association of IDO Expression with CD8^+^ TILs

**Figure 1:**
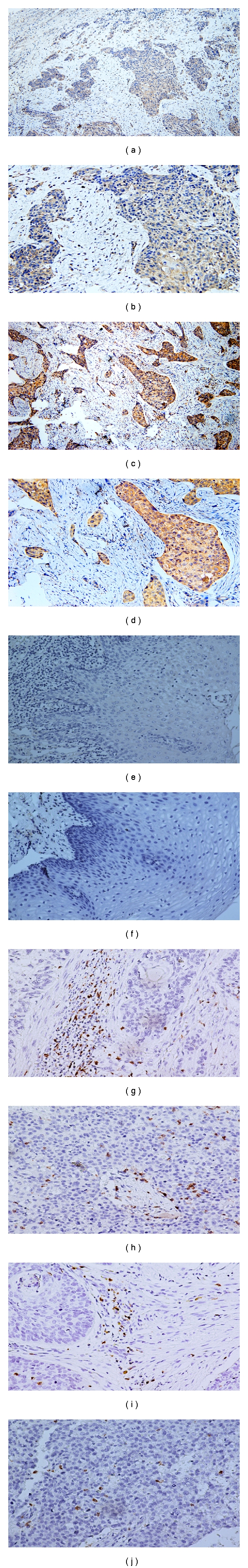
Expression analysis of IDO protein in ESCC by immunohistochemistry and representative immunohistochemical staining for CD8^+^ TILs. (a, b) Example of IDO-low expression. (c, d) Example of IDO-high expression. (e, f) Staining of IDO in normal esophageal epithelial tissue, (e) absent; (f) weak. (g) Example of stromal CD8^+^ TIL staining in IDO-low expression tumor tissue. (h) Example of intraepithelial CD8^+^ TIL staining in IDO-low expression tumor tissue. (i) Example of stromal CD8^+^ TIL staining in IDO-high expression tumor tissue. (j) Example of intraepithelial CD8^+^ TIL staining in IDO-high expression tumor tissue (original magnification, (a) and (c) ×100; (b, d, e–j) ×200).

IDO protein was detected in all 135 paraffin-embedded archived ESCC tissues (100%) by immunohistochemistry. IDO immunoreactivity was observed at various levels, and localization was observed in the cytoplasm of the tumor cells. By visual estimation, tumors were grouped into two categories: “IDO-high expression” and “IDO-low expression” according to a proportion and intensity score described in Methods (Figures 1(a)–1(d)). IDO was highly expressed in 68 of 135 (50.4%) tumor tissues, whereas 67 of 135 (49.6%) cases showed low IDO expression levels (Table 1). In contrast, in the normal esophageal tissue adjacent to cancers, IDO had absent to weak staining patterns (Figures 1(e)–1(f)).

**Figure 2:**
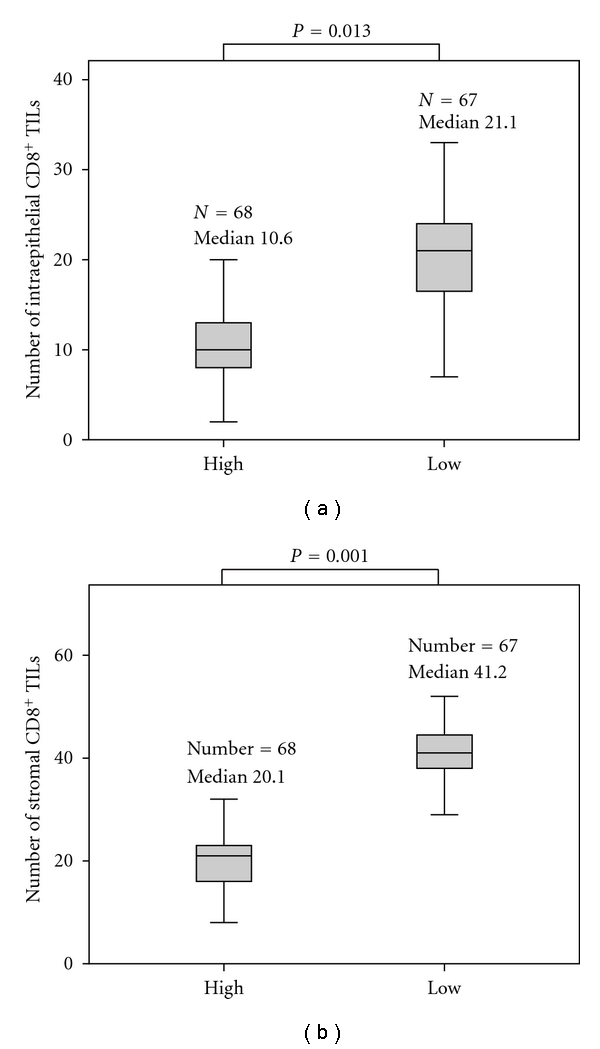
Association of IDO expression with the number of CD8^+^ TILs. There was a significant difference in both the number of intraepithelial CD8^+^ TILs (a) and stromal CD8^+^ TILs (b) between the tissues with IDO-high expression and IDO-low expression.

To investigate the relationship between IDO expression and the CD8^+^ TIL population, we evaluated the number of CD8^+^ TIL infiltrating into the tumor epithelium or stroma (Figures 1(g)–1(j)). The number of intraepithelial CD8^+^ TILs in IDO-high expressing tumors (range 1–22; median10.6) was significantly lower than in IDO-low expressing tumors (range 6–37; median 21.1; *P* = 0.013; Figure 2(a)). Similarly, IDO-high expressing tumors exhibited a significantly lower proportion of stromal CD8^+^ TILs (range 7–36; median 20.1) compared with IDO-low expressing tumors (range 18–62; median 41.2;* P *= 0.001; Figure 2(b)). The correlation of IDO expression and CD8^+^ TIL counts is summarized in Table 1. These results suggest that the level of IDO expression is inversely correlated with the number of intraepithelial CD8^+^ TILs and the number of stromal CD8^+^ TILs.

### 3.2. Association of IDO Expression with Clinicopathological Variables

As shown in Table 1, there was no significant correlation between the expression level of IDO protein and gender, age, histological classification, histological differentiation, tumor diameter, depth of invasion, and distant metastasis of esophageal cancer patients. However, the expression of IDO is closely associated with stage of esophageal cancer patients (*P *= 0.004), T classification (*P *= 0.024), and pN classification (*P *= 0.012). Higher staging, higher T classification and lymph node metastasis correlated with higher IDO expression.

### 3.3. Impact of IDO Expression and CD8^+^ TIL Counts on Patient Overall Survival

**Figure 3:**
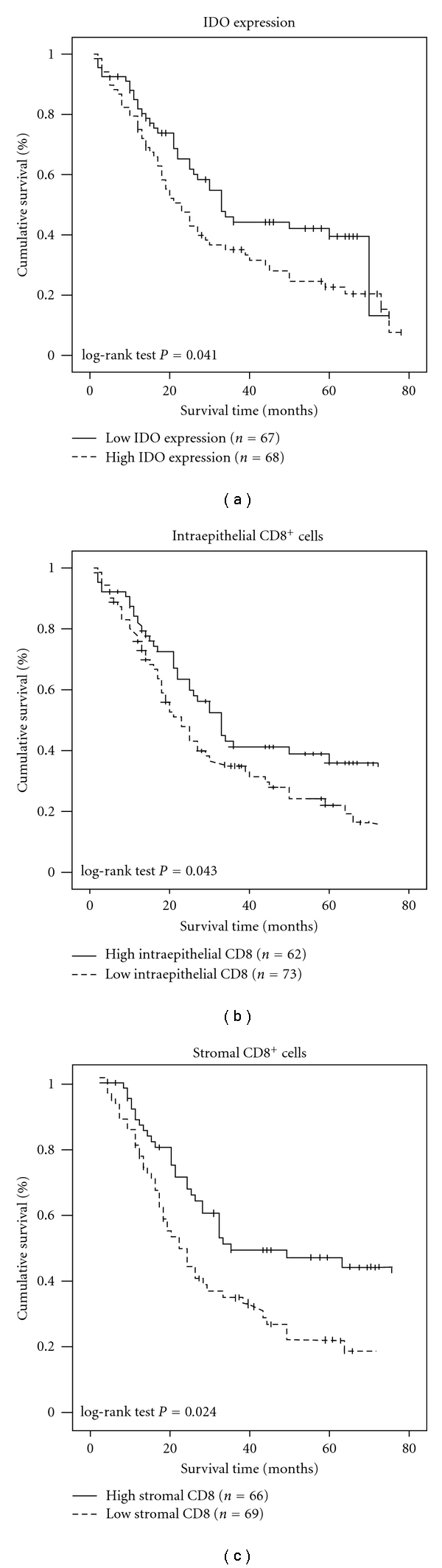
Overall survival curves drawn using Kaplan-Meier method according to the IDO expression, the number of intraepithelial CD8^+^ TILs and the number of stromal CD8^+^ TILs in 135 ESCC patients. (a) There was a significant difference in the overall survival between patients with low IDO expression (bold lines) and high IDO expression (dotted lines). (b) Significant differences in overall survival between the high intraepithelial CD8^+^ TIL groups (≥10) and the low intraepithelial CD8^+^ TIL groups (<10). (c) Significant differences in overall survival between the high stromal CD8^+^ TIL groups (≥20) and the low stromal CD8^+^ TIL groups (<20).

Overall survival analysis according to Kaplan-Meier analysis showed that although survival curves crossed at 73 months, the expression of IDO protein in esophageal carcinoma was significantly correlated with patients' survival time (*P = *0.041), indicating that higher levels of IDO expression was correlated with shorter survival time whereas the low-IDO expression group had better survival (Figure 3(a)). The median survival of patients with high IDO expression was much shorter (23 months) than those with low IDO expression (33 months).

Next, we analyzed the effect of the CD8^+^ TIL counts on patient survival.  Figure 3(b) showed that patients in the high intraepithelial CD8^+^ TIL groups (≥10) showed a significantly higher survival time compared with those in the low intraepithelial CD8^+^ TIL groups (<10; *P * = 0.043). Similarly, those in the high stromal CD8^+^ TIL groups (≥20) exhibited a significantly higher survival time compared with those in the low stromal CD8^+^ TIL groups (<20; *P *= 0.024; Figure 3(c)). These results indicated that patients with low intraepithelial and stromal CD8^+^ TILs had significantly impaired survival compared with patients with high intraepithelial and stromal CD8^+^ TILs.

### 3.4. IDO Expression in Esophageal Carcinoma Cell Lines and Induction by IFN*γ*

**Figure 4:**
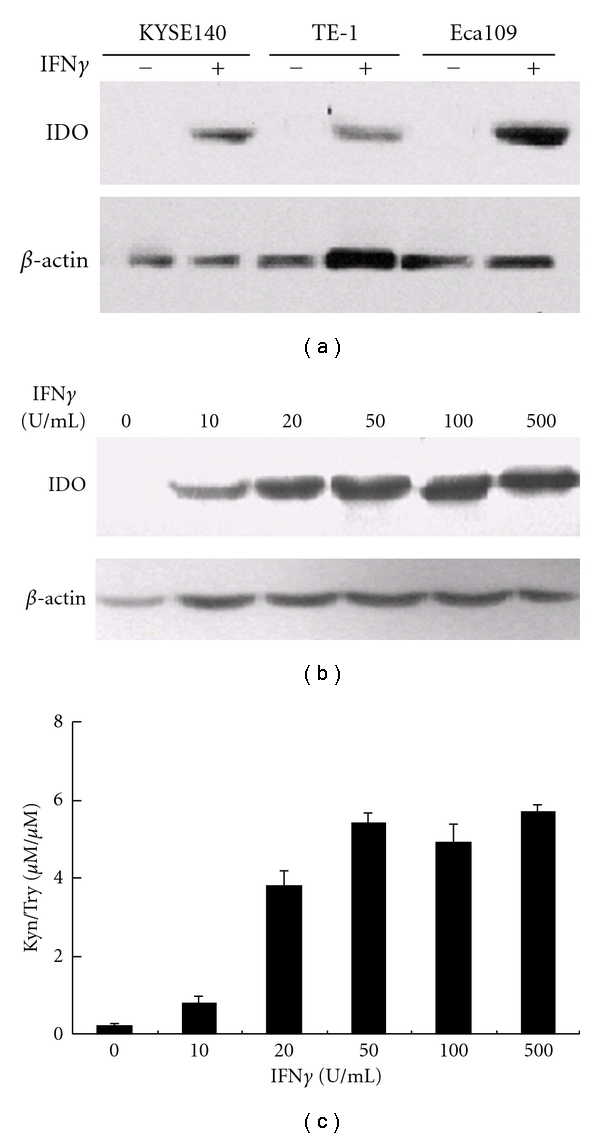
Effect of IFN*γ* on IDO expression in the esophageal carcinoma cell lines. Esophageal carcinoma cell lines Kyse 140, TE-1, and Eca109 were treated with or without 100 U/mL IFN*γ* for 24 hr, and expression of the IDO protein was assessed by western blot (a). IFN*γ* induced IDO protein expression in Eca109 cells in a dose-dependent manner. Eca109 cells were cultured in the presence of indicated concentrations of IFN*γ* for 24 hr, IDO expression was analyzed by western blotting (b); the supernatants of Eca109 cells were analyzed for Kyn and Trp production using HPLC (c). Columns, mean of three independent experiments. Bars, SD.

The effect of IFN*γ* on IDO expression was investigated in the ESCC cell lines: Eca109, TE-1, and Kyse140. As shown in Figure 4(a), the western blot assay showed that none of the cell lines constitutively expressed the IDO protein. IDO could be induced in these cell lines by treatment with 100-U/mL IFN*γ*. Among these cell lines, Eca109 had the highest expression level. Then, we performed western blot analysis to investigate the effects of varying concentrations of IFN*γ* on the expression of IDO in Eca109 cells.  Figure 4(b) shows that treatment with low-dose IFN*γ* (10 U/mL) could induce IDO expression, which was further increased in an IFN*γ* concentration-dependent manner. The enzymatic activity of IDO was also investigated by HPLC (Figure 4(c)). The enzymatic activity of IDO was undetectable in the culture medium of untreated Eca109 cells, but the activity was observed with 10 U/mL IFN*γ* stimulation, peaking with 50 U/mL IFN*γ*, and then it remained at almost the same level with increasing IFN*γ* stimulation. Thus, consistent with the results of the western blot analysis, IDO expression in ESCC cell lines was an inducible event that was highly sensitive to IFN*γ* stimulation.

### 3.5. Exposure to the Microenvironment Created by IDO-Positive Eca109 Cells Severely Suppresses CD8^+^ T  Cells Proliferation and Does Not Induce CD8^+^ T  Cell Apoptosis

**Figure 5:**
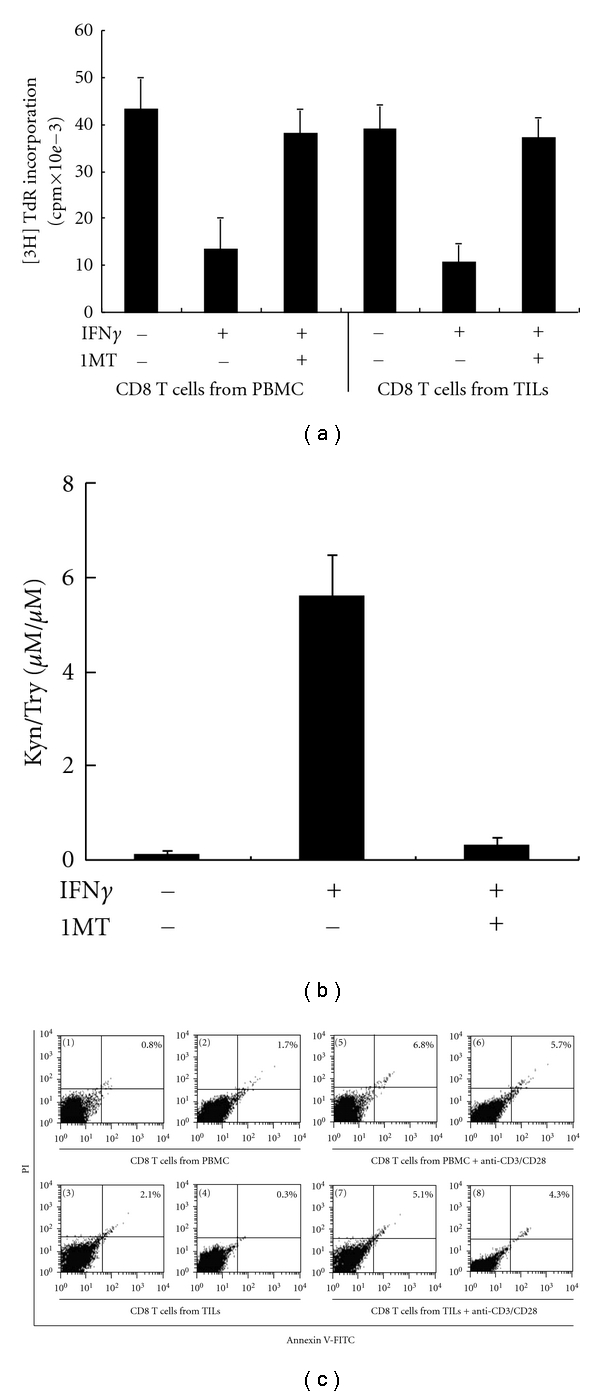
Effect of IDO on the proliferation and apoptosis of CD8^+^ T cells from both PBMCs and TILs. (a) Proliferation of CD8^+^ T cells from PBMCs or TILs was suppressed by IDO-expressing Eca109 cells. CD8^+^ T cells were cultured in conditioned media and activated with anti-CD3/CD28 antibodies. Proliferation of CD8^+^ T cells was assayed by [3H] TdR incorporation. (b) Concentrations of Kyn and Try were measured in the supernatants of Eca109 cells treated with or without IFN*γ* (50 U/mL) and/or 1MT (100 *μ*M) for 24 hr. Kyn and Try productions were analyzed by HPLC. (c) Effect of IDO on the apoptosis of CD8^+^ T cells from PBMCs or TILs. CD8^+^ T cells (1–4) or anti-CD3/CD28-activated CD8^+^ T cells (5–8) from both PBMCs and TILs were cultured in conditioned media derived from IFN*γ*-treated (2, 4, 6, 8) or untreated (1, 3, 5, 7) Eca109 cells for 4 d, and the population of apoptotic cells was detected by flow cytometric analysis, using Annexin V and Propidium Iodide as indicators. Columns, mean of three independent experiments. Bars, SD. *P < 0.05 compared with CD8^+^ T cells cultured in conditioned media derived from non-IFN*γ*-treated Eca109 cells.

To address our above observations that the samples with a high IDO expression also had a low number of CD8^+^ TILs, we tested whether the exposure to CM from IFN*γ*-treated Eca109 cells could inhibit CD8^+^ T-cell proliferation and/or induce CD8^+^ T-cell apoptosis. We treated Eca109 cells with or without IFN*γ* before CD8^+^ T cells were cultured in these CMs under anti-CD3/CD28 mAb stimulation. CD8^+^ T-cell proliferation both from PBMCs and TILs was significantly lower in treated media compared with the proliferation in untreated media (Figure 5(a)). These findings indicated that the CM derived from IFN*γ*–treated Eca109 cells suppressed CD8^+^  T-cell proliferation. Next, we tested whether the observed effects of the CM on CD8^+^ T-cell proliferation rates were related to functional IDO enzyme activity. As shown in Figure 5(b), IFN*γ*–treated Eca109 cells had IDO enzyme activity whereas untreated cells did not have IDO enzyme activity. To further demonstrate the effect of IDO on the proliferation of CD8^+^T cells, the specific IDO inhibitor 1MT was used to block the enzyme activity. When exposed to IFN*γ*-treated Eca109-CM, the proliferation of CD8^+^ T cells both from PBMCs and TILs was almost completely restored by the 1MT addition (Figure 5(a)), the functional IDO enzyme activity in IFN*γ*-treated Eca109-CM was dramatically inhibited by the 1 MT (Figure 5(b)). Taken together, the data indicated that the media created by IDO-positive Eca109 cells suppressed CD8^+^ T-cell proliferation *in vitro*.

We next aimed to determine the effects of IDO on apoptosis of CD8^+^ T cells. Without simulation, CD8^+^ T cells from both PBMCs and TILs exhibited no significant difference in the frequency of apoptotic cells between the cells exposed to IFN*γ*-treated and untreated Eca109-CM. Similarly, under anti-CD3/CD28 simulation, there was no significant difference in the frequency of apoptotic cells between the cells exposed to IFN*γ*-treated and untreated Eca109-CM (Figure 5(c)). These results indicated that IDO derived from IFN*γ*-treated Eca109 cells exerted no significant impact on the apoptosis of CD8^+^ T cells.

### 3.6. Exposure to Conditioned Medium Derived from IDO-Positive Eca109 Cells Impairs Cytolytic Activity of CD8^+^ T Cells

**Figure 6:**
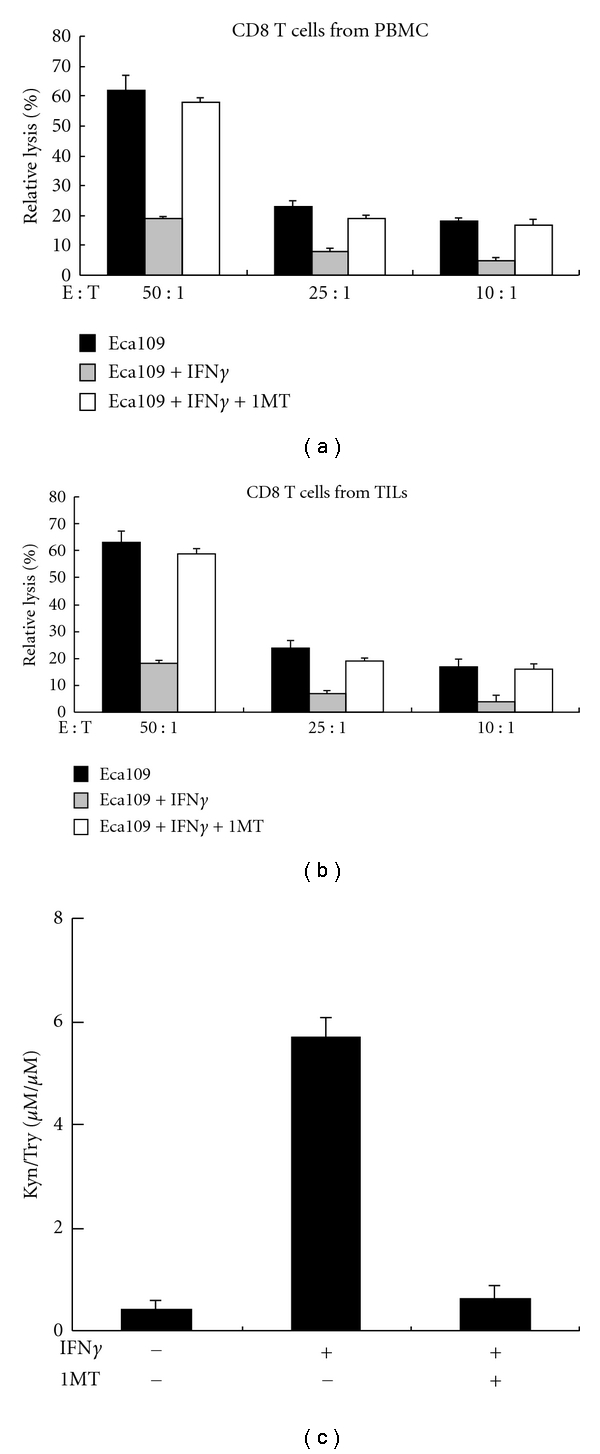
Cytolytic activity of CD8^+^ T cells from both PBMCs and TILs was impaired by incubation with media created by IDO-positive Eca109 cells. Activated CD8^+^ T cells from both PBMCs (a) and TILs (b) were cultured with CM from Eca109 cells treated with or without 50-U/mL IFN*γ* and/or 100-*μ*M 1 MT for 24 hr, and cytolytic activity against Eca109 cells was evaluated using a standard LDH release assay. The E/T ratios are indicated. (c) Eca109 cells were treated with or without 50-U/mL IFN*γ* and/or 100-*μ*M 1 MT for 24 hr. The Eca109 cell supernatants were analyzed for Kyn/Try production by HPLC. Columns, mean of three independent experiments. Bars, SD. *P < 0.05 compared with CD8^+^ T cells cultured in conditioned media derived from non-IFN*γ*-treated Eca109 cells.

To investigate whether IDO has an effect on the cytolytic activity of CD8^+^ T cells, a standard LDH release assay was conducted using Eca109 cells as targets and CD8^+^ T cells from PBMCs or from TILs stimulated by IL-2 as effectors. IL-2 activated CD8^+^ T cells from both PBMCs and TILs lysed the target cells at different E/T ratios when exposed to CMs from untreated Eca109 cells whereas the lysis rate was remarkably reduced when the T cells were exposed to IFN*γ*-treated Eca109-CMs. But the cytolytic activity of CD8^+^ T cells from both PBMCs and TILs was effectively restored when exposed to CM derived from IFN*γ*-treated Eca109 cells and in the presence of 1MT (Figures 6(a) and 6(b)). Because the IDO enzyme activity only presented in CM from IFN*γ*-treated Eca109 cells, but not in CM from untreated cells or dramatically reduced in CM from IFN*γ*-treated Eca109 cells with 1MT (Figure 6(c)). These results suggested that the inhibition on CD8^+^ T cell-mediated cytotoxicity was attributed to IDO created by IFN*γ*-treated Eca109 cells, and 1MT can abrogate this effect, providing a potential role of IDO in impairing CD8^+^ T-cell cytotoxic function.

## 4. Discussion

In this study, we confirmed previous reports that ESCC patients with higher numbers of CD8^+^ TILs within either tumor epithelium or tumor stroma had a better prognosis than those with a lower number of CD8^+^ TILs [6, 7]. Recent studies have suggested that tumoral IDO expression correlates with a reduced number of CD8^+^ T-cell infiltration into tumor sites [8–10]. In line with these observations, we also found that IDO expression in ESCC was inversely correlated with the number of CD8^+^ TILs both in tumor epithelium and tumor stroma. Moreover, our data indicated that the expression of IDO correlated with the poor clinical outcome of ESCC patients and are consistent with a previous study that ESCC patients with higher levels of IDO expression had a worse survival rate than patients with lower levels of IDO expression [24]. However, these results were in contrast with those of Liu et al. in that IDO expression was not significantly correlated with clinical outcomes [25]. The less marked impact of IDO on clinical outcomes reported by Liu et al. might be due to the comparatively small patient population analyzed. Consistent with other studies that serum/plasma Kyn/Trp levels were used as indicator of IDO activity and were found higher IDO enzyme activity predicted worse survival of cancer patients [8, 30]. Our findings also suggest that higher tumoral IDO expression and lower numbers of CD8^+^ TILs might contribute to a worse survival in ESCC patients. 

IDO is widely distributed in mammals and is preferentially inducible by IFN*γ* [11]. There have been studies indicating that IDO is upregulated in many tumor cell lines only upon treatment with IFN*γ* and/or other inflammatory mediators [31, 32]. Recently, Godin-Ethier reported that activated T cells induce functional IDO expression in breast and kidney tumor cell lines and that this was partly attributable to IFN*γ* [13]. In the present study, an analysis of Kyn production demonstrated that IDO enzymatic activity was only present in the esophageal cancer cell lines treated with IFN*γ*, and western blot also confirmed the finding. Furthermore, we showed that IFN*γ* induced the IDO expression in Eca109 cells at concentrations as low as 10 U/mL, implying that IDO expression in Eca109 cells could be easily induced by low levels of IFN*γ*. The finding that ESCC tumor cell lines show no constitutive IDO expression and that only IFN*γ* was able to induce huge activity corresponds very well to early Werner-Felmayer's study [33], but is a little against claims made by Uyttenhove [12]. This discrepancy may be the different tumor cell lines used in different studies. 

Tumor-associated antigen-presenting cells, such as macrophages and dendritic cells, and tumor-associated antigen-specific T-cells within the tumor microenvironment release this cytokine [34]; thus, they might induce IDO expression in esophageal tumor cells* in vivo*.

Once expressed in tumor cells, IDO degrades the essential amino acid Try to form N-formyl Kyn and produces a series of immunosuppressive Try metabolites [12]. Two apparently disparate mechanisms have been proposed to explain how IDO plays a role in immune suppression. One suggests that depleting local L-Try, an essential amino acid for T-cell proliferation, may block the cell cycle in the G1 phase and render T cells susceptible to proliferation arrest [12, 35, 36], and the other suggests that IDO may suppress T-cell responses by the action of Try metabolites, such as Kyn, which are toxic and proapoptotic for T cells [19]. Our data showed that exposure to the microenvironment created by IDO-positive Eca109 cells severely suppressed CD8^+^ T-cell proliferation and did not significantly induce CD8^+^ T-cell apoptosis, and the results favor the model describing the proliferation arrest of CD8^+^ T cells. Thus, the inhibition of CD8^+^ T cell proliferation locally by IDO expression in ESCC tumor cells may contribute to the high IDO expression correlated with low numbers of CD8^+^ TILs both in the tumor epithelium and tumor stroma. 

In addition, we presented the evidence that exposure to IDO-expressing Eca109-CM dramatically weakened the cytolytic function of CD8^+^  T cells from both PBMCs and TILs against target cells, but this attenuation could be abrogated by the addition of the IDO inhibitor 1 MT. In line with our observations, Liu et al. very recently reported that in the experimental rat lung allograft model, IDO creates a local microenvironment that leads to not only reduction in the numbers of CD8^+^  TILs, but also the loss of cytotoxic activity of the CD8^+^ effector T cells toward target cells [22]. The impaired cytotoxic function seen in the IDO-treated CD8^+^ T cells was accompanied by defects in the production of granule cytotoxic proteins, including perforin and granzyme A and B. Moreover, IDO leads to an impaired bioenergetic condition in active CD8^+^ T cells via selective inhibition of complex I in the mitochondrial electron transfer chain [22]. Our previous study also showed that exposure to the milieu created by IDO-positive nasopharyngeal cancer cells significantly impaired lymphocytes against target tumor cells [23]. These findings, together with our observations, suggest that IDO creates an immune suppression microenvironment not only by suppressing the proliferation of CD8^+^ TILs but also by impairing the cytotoxic function of CD8^+^ TILs. However, further studies are needed to elucidate the exact mechanisms of how IDO expressed by ESCC tumor cells reduces the cytotoxicity of CD8^+^ T cells.

In conclusion, IDO expression in ESCC correlated with the reduced number of CD8^+^ TILs, which is associated with disease progression and worse clinical outcome, may largely be due to the IDO-mediated proliferation arrest of CD8^+^ TILs. Moreover, the CD8^+^ T cells exposed to the milieu generated by IDO-expressing Eca109 cells lost their cytolytic function. We suggest that the effect of IDO expressed in ESCC cells on the proliferation and cytolytic function of CD8^+^ TILs could contribute to the finding that patients with higher IDO expression have more aggressive disease progression and a shorter overall survival time. Although the precise role of tumoral IDO in human ESCC remains to be elucidated, our findings suggest that blocking IDO activity may provide a potential means of restoring the host antitumor immunity in the treatment of ESCC.

## Figures and Tables

**Table 1:** Characteristics of 135 patients with esophageal squamous cell carcinoma and correlation between the clinicopathologic features and expression of IDO.

Characteristics	All cases (*n*)	IDO low* n* ( %)	IDO high* n* ( %)	Significance (*P*)*
Gender				
Male	100	45 (45.0)	55 (55.0)	0.069
Female	35	22 (62.9)	13 (37.1)	
Age (y)				
<60	80	35 (43.8)	45 (56.2)	0.100
≥60	55	32 (58.2)	23 (41.8)	
Stage				
I-II	74	45 (60.8)	29 (39.2)	0.004
III-IV	61	22 (36.1)	39 (63.9)	
Histological differentiation				
Well	41	25 (61.0)	16 (39.0)	0.075
Moderate	57	22 (38.6)	35 (61.4)	
Poor	37	20 (54.1)	17 (45.9)	
Tumor diameter				
<40mm	54	25 (46.3)	29 (53.7)	0.527
≥40mm	81	42 (51.9)	39 (48.1)	
Depth of invasion				
Submucosa	7	5 (71.4)	2 (28.6)	0.300
Muscularis propria	46	25 (54.3)	21 (45.7)	
Adventitia	82	37 (45.1)	45 (54.9)	
pT classification				
T1-T2	44	28 (63.6)	16 (36.4)	0.024
T3-T4	91	39 (42.9)	52 (57.1)	
pN classification				
Yes	65	25 (38.5)	40 (61.5)	0.012
No	70	42 (60.0)	28 (40.0)	
P metastasis				
Yes	9	4 (44.4)	5 (55.6)	0.982
No	126	63 (50.0)	63 (50.0)	
Intraepithelial CD8^+^				
High (≥10)	62	38 (61.3)	24 (38.7)	0.013
Low (<10)	73	29 (39.7)	44 (60.3)	
Stromal CD8+				
High (≥20)	66	42 (63.6)	24 (36.4)	0.001
Low (<20)	69	25 (36.2)	44 (63.8)	
